# Defective Spermatogenesis in Cryptorchid Testes: Cause or Effect?

**DOI:** 10.1289/ehp.11489

**Published:** 2008-08

**Authors:** Hari Prasad

**Affiliations:** School of Medical Science and Technology, Indian Institute of Technology, Kharagpur, India E-mail: haribhata@yahoo.com

Martin et al. (2008) recently published their quantitative meta-analysis focusing on the estrogen hypothesis of testicular dysgenesis syndrome. I congratulate the authors on their thorough review and excellent summary of the existing literature. The study findings are in line with other articles; however, there are several concerns that need further attention.

Martin et al. (2008) pointed out that a common etiology underlies impaired spermatogenesis, male reproductive tract abnormalities such as hypospadias and cryptorchidism, and testicular cancer. I am especially interested in exploring the relationship between defective spermatogenesis and cryptorchidism.

Maldescended testes is commonly cited as an important cause for defective spermatogenesis (Tomomasa et al. 2002). In contrast, testicular ascent (acquired cryptorchidism) could also be a risk factor for spermatogenesis in infertile men without any history of maldescended testes (Mieusset et al. 1997). However, it remains controversial whether impaired testicular function and spermatogenesis imparts an increased risk—and therefore represents a common pathogenetic mechanism of both congenital and acquired cryptorchidism—or is merely associated with disease. Recently, a potential link was proposed relating spermatogenesis and testicular descent (Skandhan and Rajahariprasad 2007). Observational studies of many lower animals (rodents, bats, and insectivores) have revealed that testicular position is dependent on its functional status: It is scrotal during breeding seasons and inguinal or abdominal at other times (Bannister and Dayson 1995). Therefore, it is possible that maldescended testes or acquired testicular ascent simply report a state of defective testicular function and spermatogenesis. In animal studies, estrogen has been shown to increase the number of type A spermatogonia, together with inhibition of their differentiation into further steps (Kula et al. 1997). Furthermore, supportive evidence suggests that undifferentiated type A spermatogonia are the only germ cells present in cryptorchid testes (Nishimune et al. 1978). I believe that the results of Martin et al. (2008) would have been more convincing if the authors could have shown that high levels of estrogens suppress spermatogenesis.

The data of Martin et al. (2008) do not allow us to extrapolate whether exposure to environmental chemicals and pollutants with estrogenic or antiandrogenic effects can cause testicular “ascent” (Barthold and González 2003). There is strong experimental evidence that prenatal exposure to environmental chemicals, including phthalate esters, is associated with an increased risk of postnatal cryptorchidism (Imajima et al. 1997). The similarity in the histopathology of the ascending testis and the testis undescended since birth suggests that ascending testes are not retractile testes trapped in scar tissue (Rusnack et al. 2002). Furthermore, this finding also suggests that, as in primary undescended testes, estrogen/antiandrogen hypotheses could explain the cause of ascending testes, because a thermal effect cannot be blamed for the decreased germ cell count in the descended testis.

Overall, the systematic review and meta-analysis by Martin et al. (2008) is the most extensive attempt to date to investigate the link between estrogenic agents and testicular dysgenesis syndrome. Although some of the data from the cited studies are of limited quality, the fact that nearly all of the included studies identified an increase in the risk of hypospadias, cryptorchidism, and testicular cancer in the groups prenatally exposed to diethylstilbestrol provides strong support for that association being genuine. However, from the data of Martin et al. (2008), we cannot conclude whether exposure to environmental chemicals with estrogenic effects significantly increases the risk of developing acquired cryptorchidism. Further research to evaluate the effects of endocrine-disrupting chemicals (EDCs)—particularly those with estrogen-like effects on reproductive health—is justified and should continue.
